# Airway Microbiome Is Associated with Atherosclerosis in Adults from Southern Caribbean

**DOI:** 10.3390/microorganisms14081699

**Published:** 2026-08-02

**Authors:** Diana Mena-Yi, Alejandra Puerto, Sara Mestra, Josefina Zakzuk, Marlon Munera, Fernando Manzur-Jattin, Maria S. Ruiz-Diaz, Gustavo Mora-Garcia

**Affiliations:** 1 Laboratory of Research and Development in Public Health, Research Department, Faculty of Medicine, Universidad de Cartagena, Cartagena 130015, Colombia; dmenay@unicartagena.edu.co (D.M.-Y.); mruizd2@unicartagena.edu.co (M.S.R.-D.); 2 Microbiology Master Program, Graduate Department, Faculty of Medicine, Universidad de Cartagena, Cartagena 130015, Colombia; 3 Institute for Immunological Research, University of Cartagena, Cartagena 130015, Colombia; apuertol@unicartagena.edu.co (A.P.); jzakzuks@unicartagena.edu.co (J.Z.); 4 Grupo de Investigación Genoma, Universidad del Sinú, Elías Bechara Zainúm, Seccional Cartagena, Cartagena 130014, Colombia; 5 Biology Program, Faculty of Sciences, Universidad de Cartagena, Cartagena 130015, Colombia; smestrab@unicartagena.edu.co; 6 Basic Science Department, Faculty of Medicine, Corporación Universitaria Rafael Nuñez, Cartagena 130001, Colombia; marlon.munera@curn.curnvirtual.edu.co; 7 Physiology Unit, Basic Science Department, Faculty of Medicine, Universidad de Cartagena, Cartagena 130015, Colombia; fmanzurj@unicartagena.edu.co; 8 Center for Innovation and Research on Metabolism and Diabetes—INNOVATID, Cartagena 130001, Colombia

**Keywords:** microbiome, upper respiratory tract, atherosclerosis, Caribbean region, upper respiratory infection

## Abstract

Subclinical atherosclerosis (SCA) has been associated with lung function in many populations. Accordingly, those factors involved in respiratory health may contribute to the mechanisms linking pulmonary and cardiovascular disorders. Since the airway microbiome plays a key role in respiratory pathophysiology, microbial abundance and diversity in the upper airway may be associated with SCA risk. This study aimed to assess the association of upper airway microbiome abundance and diversity with SCA risk. A nested case–control study was carried out among adults from the Southern Caribbean. SCA was defined by carotid Doppler ultrasound as a carotid intima-media thickness ≥ 0.8 mm or the presence of carotid plaque. Oropharyngeal mucosal samples were collected under fasting conditions, and the V3-V4 region of the *16SRNA* gene was sequenced. Differences in genus-level abundance were assessed using Microbiome Multivariable Association with Linear Models 2 (MaAsLin2). Alpha-diversity indices were compared through Wilcoxon tests. Principal coordinates analysis (PCoA) and Permutational Multivariate Analysis of Variance (PERMANOVA), adjusted for age, body mass index, and smoking status, were performed to assess associations between Bray–Curtis distances and SCA. MaAsLin2 was also applied to determine predicted metabolic pathway enrichment. A total of 40 cases and 40 controls were analyzed. The oropharyngeal microbiomes composition was dominated by three genera: *Prevotella* (cases: 26.0%; controls: 29.8%), *Veillonella* (cases: 16.3%; controls: 13.7%) and *Fusobacterium* (cases: 7.5%; controls: 8.0%). Significant differences in relative abundance were found for *Streptococcus*, *Aureimonas*, *Mogibaecterium*, *Candidatus Saccharimonas*, *Pseudomonas*, *Segatella*, *Stomatobaculum*, *Oribacterium* and *Lachnoanaerobaculum*, with lower abundance in cases for all these genera. Alpha-diversity was lower in cases than in controls, as reflected by the Shannon index (cases: 5.95 IQR [4.87; 6.35] vs. controls: 6.49 IQR [6.29; 6.75], *p* < 0.001), Simpson index (cases: 0.994, IQR [0.989; 0.996] vs. controls: 0.997 IQR [0.997; 0.998], *p* < 0.001) and Chao1 richness (cases: 1076.2 IQR [345.2; 1473.4] vs. controls: 1136.1, IQR [841.1; 1635.3], *p* = 0.04) were compared. In adjusted-PERMANOVA, 8.2% of microbiome variations were associated with SCA (*pseudo-F* = 7.49, *R*^2^ = 0.082, *p* < 0.001), although this finding may have been influenced by intra-group dispersion among cases (PERMDISP: *pseudo-F* = 5.62, *p* = 0.018; Tukey’s HSD test: mean difference = −0.071, 95% CI [−0.118, −0.025], *p* = 0.003) Predicted enrichment of phenylalanine metabolism and indole alkaloid biosynthesis was higher in cases. In conclusion, these exploratory analyses suggest that reduced oropharyngeal microbiome diversity is associated with SCA. Further studies are warranted to clarify the role of the airway microbiome on cardiovascular outcomes.

## 1. Introduction

Subclinical atherosclerosis (SCA) plays a central role in the development of cardiovascular diseases (CVDs) [1]. Atheroma destabilization typically precedes life-threatening cardiovascular events, including stroke, ischemic coronary artery disease, and other major complications [2,3]. Atherosclerotic cardiovascular disease (aCVD) is one of the leading causes of mortality worldwide [4], accounting for more than 7.7 million deaths annually and 16.8% of all adult deaths [5,6]. The attributed morbidity is also substantial, accounting for 40.8 million disability-adjusted life years (DALYs) and 36.4 million years of life lost due to premature mortality [7].

SCA trajectory is influenced by a wide range of modifiable and non-modifiable risk factors. These factors commonly converge to promote low-grade systemic inflammation and endothelial dysfunction [2,8,9,10]. In this regard, the human microbiota has emerged as a relevant contributor of aCVD and SCA [11,12]. Both gut and oral microbiomes have been associated with cardiovascular risk across many populations. In general, gastrointestinal dysbiosis is characterized by proliferation of bacteria belonging to the phyla Firmicutes/Bacillota and Proteobacteria [13]. Likewise, in oral cavity, increased relative abundance of genus of Bacillota (Firmicutes) phyla (e.g., *Prevotella*, *Megasphaera*, and *Veillonella*) has been associated with greater atherosclerosis prevalence. Conversely, a lower risk of SCA has been found to be associated with higher abundance of *Absconditabacteria*, *Capnocytophaga*, *Gemella*, *Fusobacterium*, *Neisseria*, *Aggregatibacter*, *Tannerella*, *Treponema*, *Cardiobacterium*, and *Bacteroidales*, among others [14]. This microbiota-related cardiovascular risk is thought to be mediated by metabolic shifts toward enriched biosynthesis of proinflammatory metabolites, such as trimethylamine N-oxide and lipopolysaccharides [15].

Respiratory decline is another factor associated with higher risk of SCA and CVD [16]. Although this association is often overlooked in clinical practice, accumulating evidence has consistently demonstrated an epidemiological relationship between respiratory and cardiovascular disorders [17,18]. For instance, impaired pulmonary function and chronic obstructive pulmonary disease (COPD) are known to increase up to two-fold the risk of SCA [16]. Similarly, ischemic cardiovascular events occur approximately twice as frequently following COPD exacerbations [19]. Although the underlying pathophysiological mechanisms have not been fully elucidated, current evidence suggests that dysregulated systemic oxidative stress and inflammatory responses may contribute to this association [18,20].

Substantial evidence indicates that the respiratory tract microbiota influences pulmonary inflammation and oxidative stress response [21,22,23]. In the oropharynx, a high relative abundance of *Rhodobacter* and *Prevotella* has been found to promote Th17-mediated immune responses [24]. Furthermore, interactions between the respiratory microbiota and bacteria migrating from gastrointestinal tract (e.g., *Veillonella* and *Streptococcus* spp.) induce the synthesis of proinflammatory factors, such as IL-6, IL-8, IL-10, and tumor necrosis factor-α, among others [25]. In the lower respiratory tract, members of the phyla Proteobacteria and Bacillota (Firmicutes) have been reported to play an essential role in maintaining immune homeostasis [26].

The upper airway microbiome is a dynamic community that continuously interacts with those of the nasal and oral cavities, as well as with microorganisms originating from the esophagus or stomach [27]. Nasopharyngeal and lung microbiomes exhibit considerable ecological similarity, sharing a relatively high abundance of phyla Bacteroidota, Bacillota (Firmicutes), and Proteobacteria [28]. Studies with sterile lung tissue dissection have demonstrated that this ecological continuity constitutes an unified microbial ecosystem [29], suggesting that the upper airway microbiome may serve as a proximal surrogate of the status of microbial communities colonizing the lower respiratory tract [30].

In the present study, the upper airway microbiome was analyzed under the assumption that it may reflect the architecture of microbial communities inhabiting the respiratory tract. Given that the local microbiota influences inflammatory response, we hypothesized that microbiome composition may contribute to the association between respiratory performance and the presence of SCA. Accordingly, this study aimed to assess the association between microbiome abundance and diversity with SCA risk.

## 2. Materials and Methods

A case and control study was conducted with adults living in urban areas of Southern Caribbean region. Both groups were nested within the Cartagena Cohort Study (CaReS-NCT05339048), which is designed to assess biological and socioeconomic factors of cardiovascular and respiratory health. Recruitment criteria, sampling methods, and clinical data collection procedures have been described elsewhere [31]. Briefly, a randomized cluster sampling strategy was implemented in urban areas along the Colombian Caribbean Coast. Subjects aged 18–80 years were recruited using city blocks as primary selection units (PSU), and the probability of selection was adjusted for PSU size using population density data obtained from the local housing authority. Fieldwork continued until a sample of 900 subjects was achieved. All participants provided written informed consent prior to enrollment, and the study protocol was approved by the Ethics Committee of the University of Cartagena (Act 156-2022).

Case and control groups were constructed using the CaReS baseline database while maintaining a 1:1 ratio. Cases for SCA were defined according to carotid ultrasound findings following Mannheim consensus recommendations. Thus, participants with a carotid intima-media thickness ≥ 0.8 mm or the presence of atherosclerotic plaque were classified as cases [32].

Group size was determined using a finite population formula considering the total CaReS cohort (900 subjects), a 95% confidence interval, 80% statistical power, and a hypothetical prevalence of microbiota dysbiosis of 80% among cases. This assumption was based on previous reports showing lower alpha diversity in oral and gut microbiomes associated with cardiometabolic outcomes. These parameters were applied in the equation described below.

$$N_{Kelsey}=\frac{{\left(z_{\alpha /2}+z_{\beta }\right)}^{2}p\left(1-p\right)\left(r+1\right)}{r{\left(p_{0}-p_{1}\right)}^{2}}$$

Accordingly, a sample size of 80 subjects was selected, comprising 40 cases and 40 controls. Cases and controls were identified from CaReS baseline data. Subjects were selected following a manual a manual review of baseline cardiometabolic characteristics (i.e., blood pressure, fasting glucose, triglycerides, and LDLc) to maximize comparability between groups. This procedure was not intended to achieve individual matching, but rather to reduce baseline differences between groups. Pregnant women, individuals with a previous diagnosis of autoimmune diseases, primary endocrine diseases, or cognitive or motor disabilities; and those diagnosed with cancer within 5 years before the start of the study were excluded. Participants underwent a comprehensive clinical assessment to collect data on socioeconomic variables, personal history of CVD, lifestyle factors, and anthropometric parameters. Smoking status was categorized as never, former, or current. Former smokers were defined as those who discontinued smoking for more than 12 months prior to enrollment. Lung function was measured by spirometry under pre- and post-bronchodilator conditions using a Spirolab MIR 911080 spirometer (MIR Medical International Research, Rome, Italy). Spirometric maneuvers were performed by trained physicians (D.M-Y. and M.R-D.) in accordance with the recommendations of the Global Initiative for Chronic Obstructive Pulmonary Disease Spirometry Guide.

### 2.1. Vascular Ultrasound Assessment

Bilateral carotid Doppler ultrasound examinations were performed by a single cardiologist (F.M-J.) to minimize potential operator-dependent measurement bias. The major cervical vascular structures were evaluated using a Mindray M7 color Doppler ultrasound system equipped with a 5–12 MHz linear array transducer (Mindray Bio-Medical Electronics Co., Shenzhen, China). Images of common carotid arteries, internal carotid arteries, external carotid arteries, and vertebral arteries were obtained.

### 2.2. Upper Airway Sample Collection and Microbiome Sequencing

Oropharyngeal swabs were used to collect biological samples from the mucosal surface. Sterile Dacron or polypropylene swabs were introduced through the palatal arches, avoiding contact with the uvula, cheeks, teeth, and tongue until the posterior wall of the oropharynx was reached. Each swab was rotated four to six times, placed into a 1.5 mL microtube, and immediately froze at −20 °C. Within two hours after collection, the samples were processed for nucleic acids extraction or storage at −80 °C until further usage.

Genomic DNA was extracted from oropharyngeal samples using the Saliva DNA Isolation Kit (Norgen Biotek, Thorold, ON, Canada), following the manufacturer’s instructions. Briefly, oropharyngeal mucosal swabs were immersed in a buffered solution and vortexed to release biological material into suspension. Cell lysis buffer was subsequently added, followed by proteinase K digestion and incubation to ensure efficient disruption of microbial cell walls, including those of microorganisms resistant to osmotic lysis. Nucleic acids were then purified using successive silica column-based purification steps, according to the manufacturer’s protocol. An extraction blank was included with each extraction batch to monitor potential contamination during DNA extraction and to identify false positive results. No positive extraction controls (e.g., mock microbial communities) were included at this stage of the workflow. The quality and quantity of the extracted DNA were assessed by spectrophotometry. DNA concentrations above 50 ng/µL, with purity between 1.6 and 2.0 for a 260/280 nm absorbance ratio, and between 2.0 and 2.2 for a 260/230 nm ratio, were considered acceptable. Hypervariable V3–V4 regions of the 16S ribosomal RNA (rRNA) gene were amplified through polymerase chain reaction (PCR). Primers containing adapters sequence were used to amplify target region (forward primer 5′ TCGTCGGCAGCGTCAGATGTGTATAA-GAGACAGCCTACGGGNGGCWGCAG 3′; reverse primer 5′ GTCTCGTGGGCTCGGA-GATGTGTATAAGAGACAGGACTACHVGGGTATCTAATCC 3′). Amplification was performed under manufacturer’s recommended conditions, using 10–100 ng of gDNA template per reaction and KAPA HiFi HotStart ReadyMix (Beckman Coulter, Brea, CA, USA) in a 25 µL reaction. Each PCR assay began with a pre-PCR denaturation (95 °C for 90 s), followed by 35 cycles consisting of three steps: (i) denaturation (95 °C for 30 s), (ii) primers annealing (56 °C for 30 s), and (iii) extension (72 °C for 60 s). Amplification ended with a final elongation (72 °C for 10 min).

For library preparation, PCR amplicons were purified by Solid-Phase Reversible Immobilization (SPRI), using AMPure XP Beads (Beckman Coulter, Brea, CA, USA) and appropriate buffers in a magnetic rack. Purified amplicons were subsequently indexed by PCR with the Nextera XT Index Kit (Illumina, San Diego, CA, USA). KAPA HiFi HotStart ReadyMix (Beckman Coulter, Brea, CA, USA) was used for PCR in a 50 µL reaction, which began with an initial denaturation step (95 °C for 3 min), followed by eight cycles consisting of three steps: (i) denaturation (95 °C for 30 s), (ii) primer annealing (55 °C for 30 s), (iii) extension (72 °C for 30 s); and a final extension (72 °C for 5 min).

The indexed fragments library was purified by SPRI using the AMPure XP Beads (Beckman Coulter, Brea, CA, USA). Then, the fragment size was verified by capillary electrophoresis and quantified by fluorometry, after which libraries were normalized at 70 pM. Library pools were spiked with 20% PhiX control (Illumina, San Diego, CA, USA) prior to sequencing to a minimum depth of 10,000 reads per sample on a Illumina iSeq100 platform (Illumina, San Diego, CA, USA).

Paired-end sequences were processed using the dada2 package V.1.40.0 in R-4.6.1 software (R Foundation for Statistical Computing, Vienna, Austria) [33]. Quality filtering was performed using a Phred score of ≥ 30 (Q30). Chimera removal and read pairing were performed, and the SILVA 138.2 (SSURef_NR99 uniform classifier V4-515f-806r) database was used as the reference database for taxonomic assignment at the genus and species levels.

### 2.3. Statistical Analysis

Sociodemographic and clinical variables were summarized using mean and frequencies, when appropriate. Hypothesis testing tests was performed to determine differences between cases and controls. Relative abundance and alpha and beta diversity were estimated based on microbiome composition. Microbiome Multivariable Association with Linear Models 2 (MaAsLin2) was applied to assess differences in abundance at the genus level, adjusting for age, body mass index and smoking status. The Wilcoxon rank-sum test was used to estimate differences between groups for Shannon, Simpson and Chao1 alpha diversity indexes. A principal coordinates analysis (PCoA) based on Bray–Curtis distances was executed to identify potential microbiome clustering according to SCA status, and median distance to centroid was used to assess cluster homogeneity. Permutational Multivariate Analysis of Variance (PERMANOVA) with 999 permutations, adjusted by age, body mass index and smoking status was performed to estimate the association between microbiome composition and SCA. The permutation test for homogeneity of multivariate dispersions (PERMDISP) and post hoc pairwise analysis by Tukey’s HSD test were performed to estimate within-group microbiome dispersion. Enrichment of metabolic pathways was assessed through MaAsLin2 adjusted by age, body mass index and smoking status. The normalized values were reported as z-scores. As usual, the *p* value and false discovery rate (FDR) < 0.05 were considered statistically significant. All procedures were conducted in RStudio 2025.05.0+496 for windows (Posit Software PBC, Boston, MA, USA), running the packages Biostrings V.2.80.1, dada2 V.1.40.0 [33], phyloseq V.1.56.0 [34], MaAslin2 V.1.26.0 [35] and Tax4Fun2 V.1.1.5 [36].

## 3. Results

A total of 80 subjects were included in the study (40 cases and 40 controls). Mean age was 59.7 ± 13.4 years in the case group and 44.9 ± 16.2 years in the control group (*p* < 0.001). Median monthly income was USD $250 for cases and USD $325 for controls. There were no differences between groups for the smoking status distribution (Table 1).

### Upper Airway Microbiome

**Figure 1:**
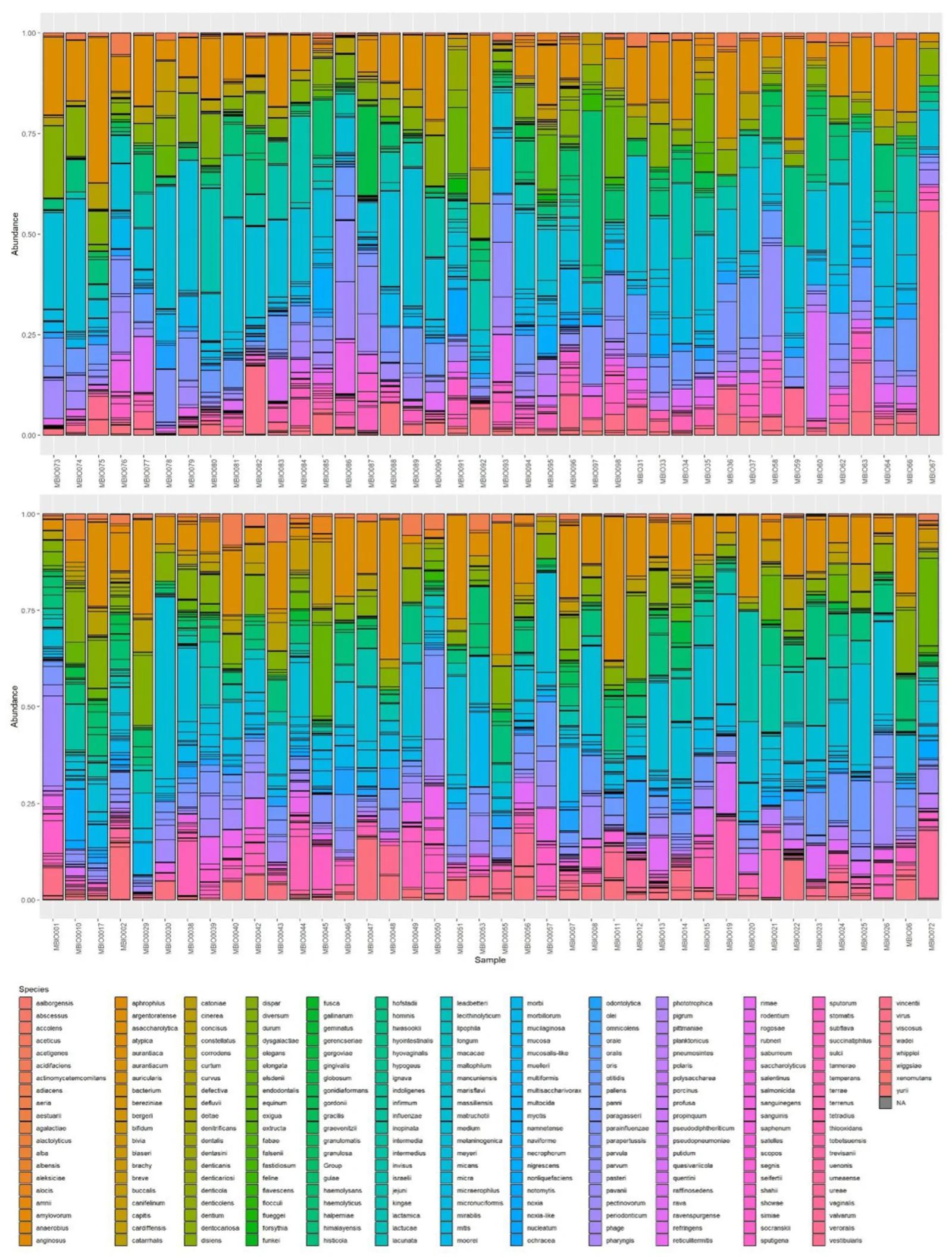
Microbiome composition based on relative abundance at the species level of the main bacterial taxa in cases (**upper** panel) and controls (**lower** panel) for subclinical atherosclerosis.

A total of 80 samples (40 cases and 40 controls) were available for sequencing analysis. After quality filtering and chimera removal, a total of 2,963,745 sequences were retained (cases: 982,980; controls: 1,980,765), with a median sequencing depth of 29,210 IQR [20,898; 56,938] (Supplementary Table S1). From these, 26,540 unique sequences were assigned to 197 genera, whereas 19,691 unique sequences achieved species-level taxonomic classification, encompassing 249 distinct taxa (Figure 1). Rarefaction curves based on sequencing depth indicated an increase in species richness with sequencing depth until curve plateau was reached (Supplementary Figure S1).

**Figure 2:**
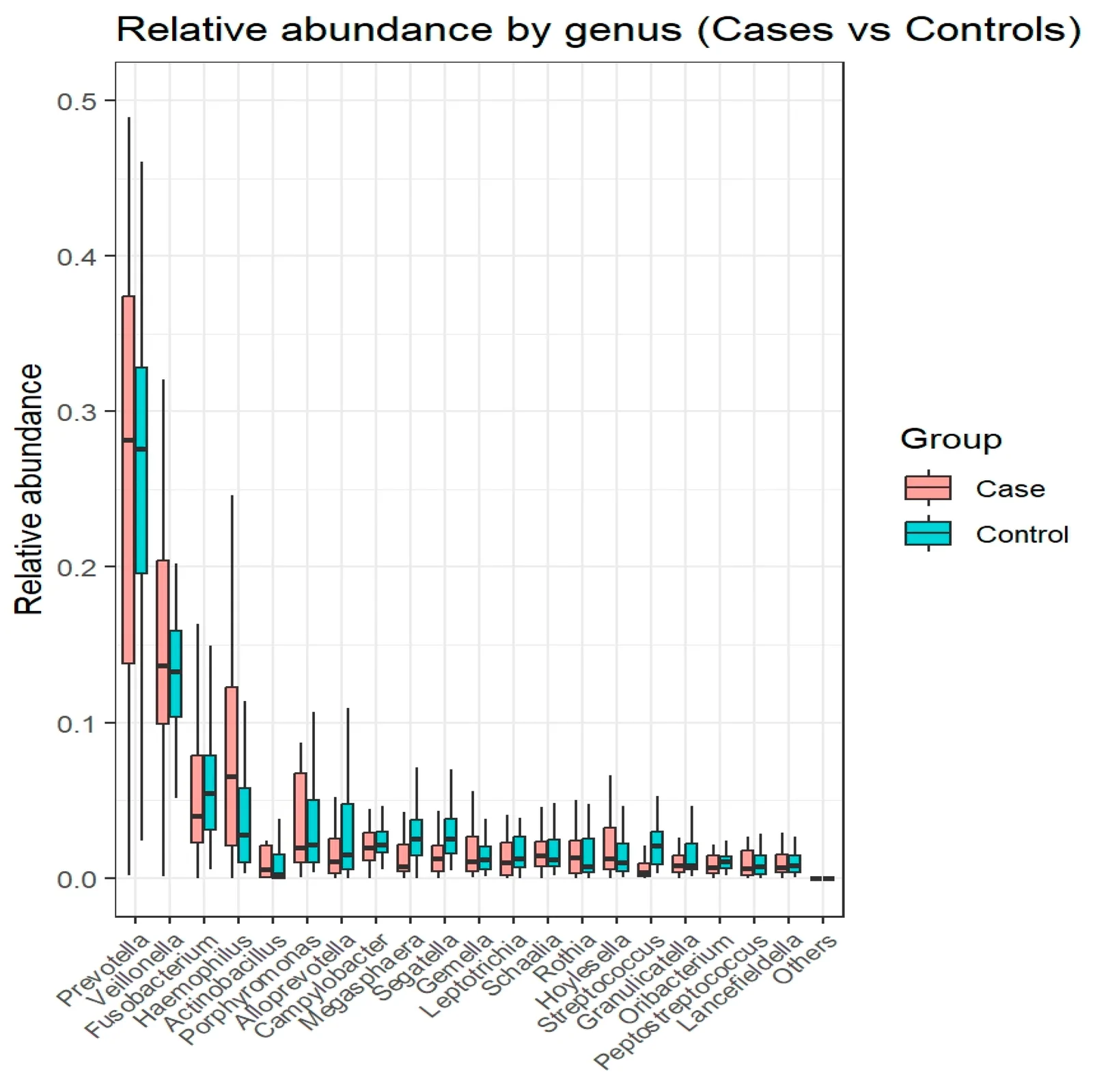
Relative abundance at genus level, classified by cases and controls for subclinical atherosclerosis. (First 20 more relatively abundant genus are represented).

Relative abundances for the most prevalent genera in cases and controls are shown in Figure 2, and a complete list of genera and species is provided in the Supplementary Materials (Supplementary Tables S2 and S3).

**Figure 3:**
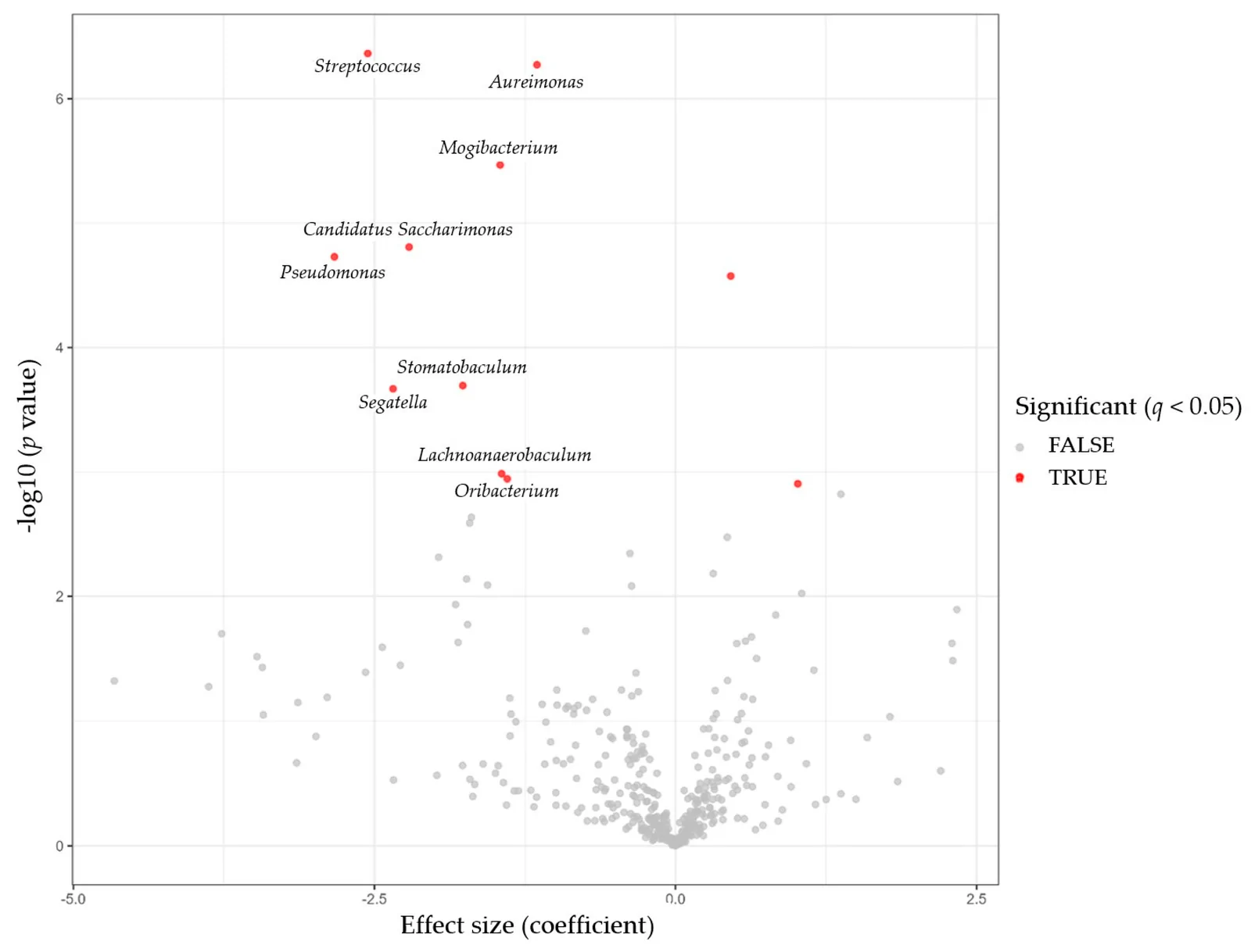
Microbiome Multivariable Association with Linear Models 2 (MaAsLin2) adjusted by age, body mass index and smoking status to assess the differences between cases and controls for relative abundance at the genus level.

At the genus level, *Prevotella* was the predominant taxon in both groups, accounting for 29.6% of sequences in cases and 30.5% in controls. *Veillonella* ranked second in cases (19.8%) but was less abundant in controls (15.2%), whereas *Fusobacterium* was more frequent in controls (6.5%) than in cases (5.1%). *Haemophilus* exhibited a higher relative abundance in cases (7.6%) compared to controls (3.4%). Conversely, *Porphyromonas* was more abundant in controls (4.9%) than in cases (3.6%). Other genera, such as *Alloprevotella*, *Actinobacillus*, *Campylobacter*, and *Leptotrichia*, contributed between 2% and 5% of the microbial community, while the remaining taxa each represented less than 2% of the total sequences (Supplementary Table S2). When adjusted-MaAsLin2 was applied, significant differences in relative abundance between groups were found for *Streptococcus*, *Aureimonas*, *Mogibacterium*, *Candidatus Saccharimonas*, *Pseudomonas*, *Segatella*, *Stomatobaculum*, *Oribacterium* and *Lachnoanaerobaculum* (Figure 3).

**Figure 4:**
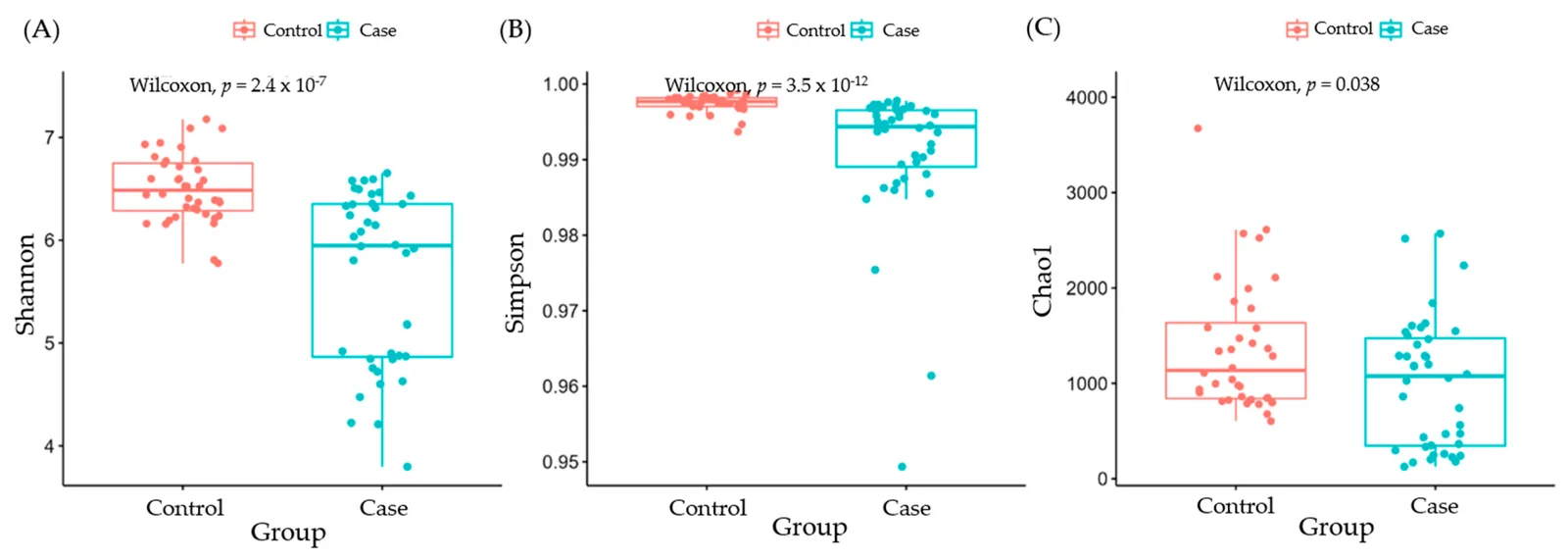
Comparison of alpha diversity indexes between cases and controls. (**A**) Simpson index; (**B**) Shannon diversity index; (**C**) Chao1 diversity index.

For alpha-diversity indices, subjects with SCA exhibited lower diversity levels, with a median Shannon index of 5.95 IQR [4.87; 6.35] compared to 6.49 IQR [6.29; 6.75] in controls (*p* < 0.001). Similarly, the Simpson index was slightly but significantly lower among cases (0.994, IQR [0.989; 0.996]) relative to controls (0.997, IQR [0.997; 0.998]) (*p* < 0.001), and Chao1 richness was also lower in cases (1076.2, IQR [345.2; 1473.4]) than in controls (1136.1, IQR [841.1; 1635.3]) (*p* = 0.038). Comparisons of alpha-diversity indices between case and control groups are shown in Figure 4.

**Figure 5:**
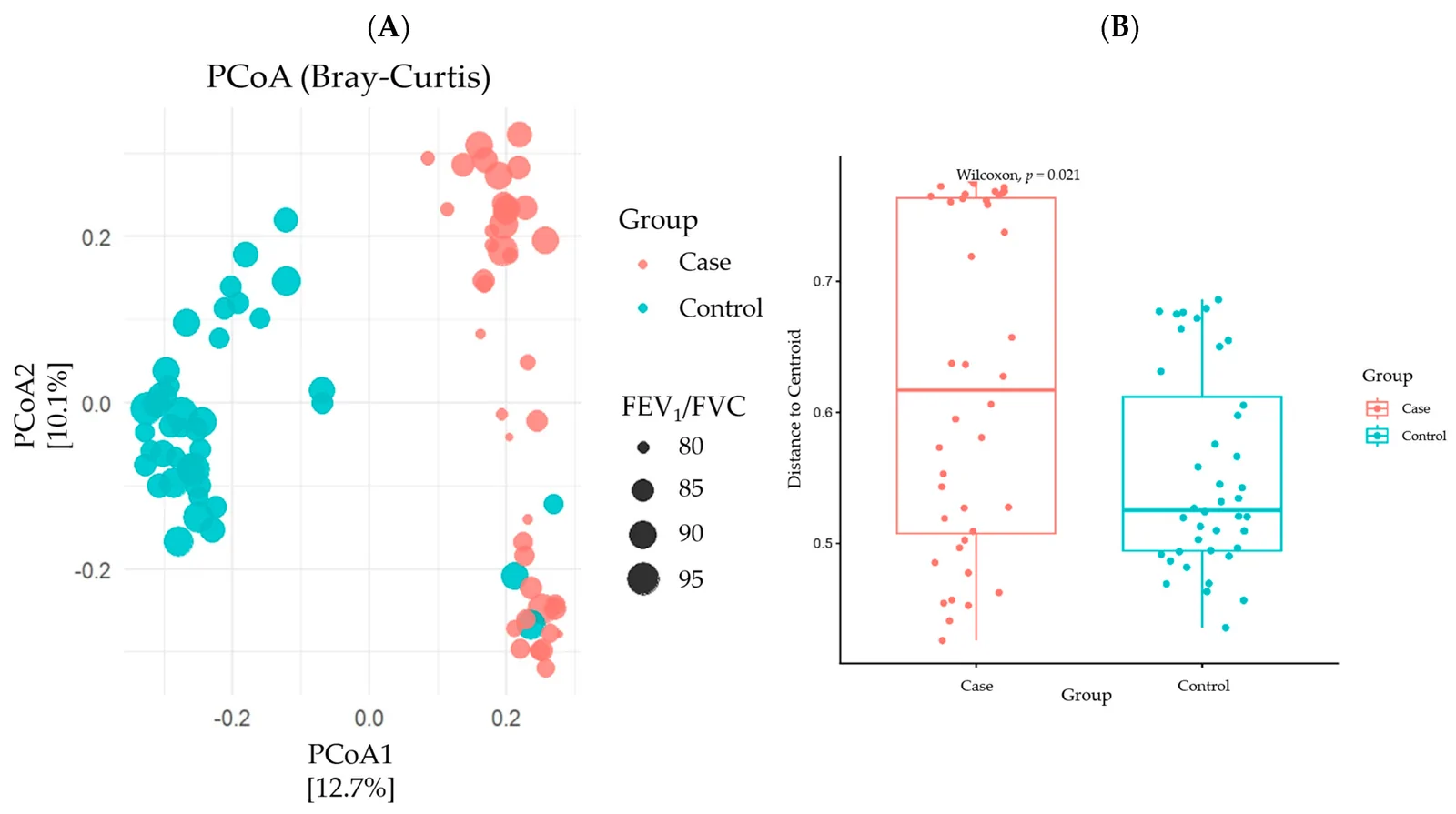
(**A**) Principal coordinates analysis based on Bray–Curtis distance for cases and controls. (**B**) Distance to centroid.

PCoA based on Bray–Curtis distances suggested a clustering of samples, with marked segregation between cases and controls (Figure 5). The median distance to the centroid was higher among cases than among controls (cases: 0.62 IQR [0.57; 0.76]), controls: 0.53 IQR [0.49; 0.61]; *p* = 0.021) (Figure 5).

When assessing differences in Bray–Curtis distance between cases and controls as a measure of beta-diversity, unadjusted PERMANOVA showed that group classification explained 12.0% of microbial community dissimilarities (*pseudo-F* = 10.60, *R*^2^ = 0.120, *p* < 0.001; 9999 permutations). After adjustment for age, sex, body mass index, smoking status, and alcohol consumption as covariates, group differences remained significant but the proportion of explained variation decreased to 8.2% (*pseudo-F* = 7.49, *R*^2^ = 0.082, *p* < 0.001). Current smoking was also independently associated with microbial community composition (*R*^2^ = 0.018, *p* = 0.017), whereas age, sex, body mass index, and alcohol consumption were not significantly associated with beta diversity.

Since the interpretation of PERMANOVA interpretation is influenced by the homogeneity of the groups, this assumption was evaluated through PERMDISP. This analysis revealed that dispersion differed significantly between groups (*pseudo-F* = 5.62, *p* = 0.018), indicating that microbial community composition was not equally homogeneous across groups. Post hoc pairwise comparisons using Tukey’s HSD test confirmed that cases had greater dispersion than controls (mean difference = −0.071, 95% CI [−0.118, −0.025], *p* = 0.003). Therefore, the PERMANOVA results should be interpreted with caution, as both centroid separation and dispersion differences may contribute to the observed effect.

An adjusted constrained distance-based redundancy analysis (dbRDA) was performed to evaluate the robustness of the primary findings. Under these conditions, group classification remained the strongest explanatory factor (*pseudo-F* = 10.9, *p* = 0.001), while smoking status was also independently associated with microbial community composition (*pseudo-F* = 0.6, *p* = 0.007). No other significant association for age, sex, BMI or alcohol consumption was observed.

As sensitivity analyses, given that age and LDLc differed between study groups at baseline, additional stratified analyses were performed to determine whether these imbalances influenced the observed beta-diversity differences. Hence, separated adjusted PERMANOVA models were carried out for subject < 60 and ≥ 60 y.o. For the younger group, the differences in beta-diversity attributable to SCA status remained significant (*pseudo-F* = 5.59, *R*^2^ = 0.114, *p* < 0.001). Similarly, in the model with the older age group, SCA remained significantly associated with beta-diversity (*pseudo-F* = 5.02, *R*^2^ = 0.128, *p* < 0.001), and smoking status was also significantly associated (*pseudo-F* = 1.53, *R*^2^ = 0.039, *p* = 0.035). Since LDLc differed between cases and controls, an additional sensitivity analysis based on dyslipidemia status (i.e., ≥ 130 mg/dL) was performed. Among participants without dyslipidemia, both SCA and smoking status were associated with microbiome beta-diversity (SCA: *pseudo-F* = 4.10, *R*^2^ = 0.089, *p* < 0.001; smoking: *pseudo-F* = 1.52, *R*^2^ = 0.033, *p* = 0.028). In contrast, among participants with dyslipidemia, only SCA remained significantly associated (*pseudo-F* = 5.24, *R*^2^ = 0.123, *p* < 0.001).

**Figure 6:**
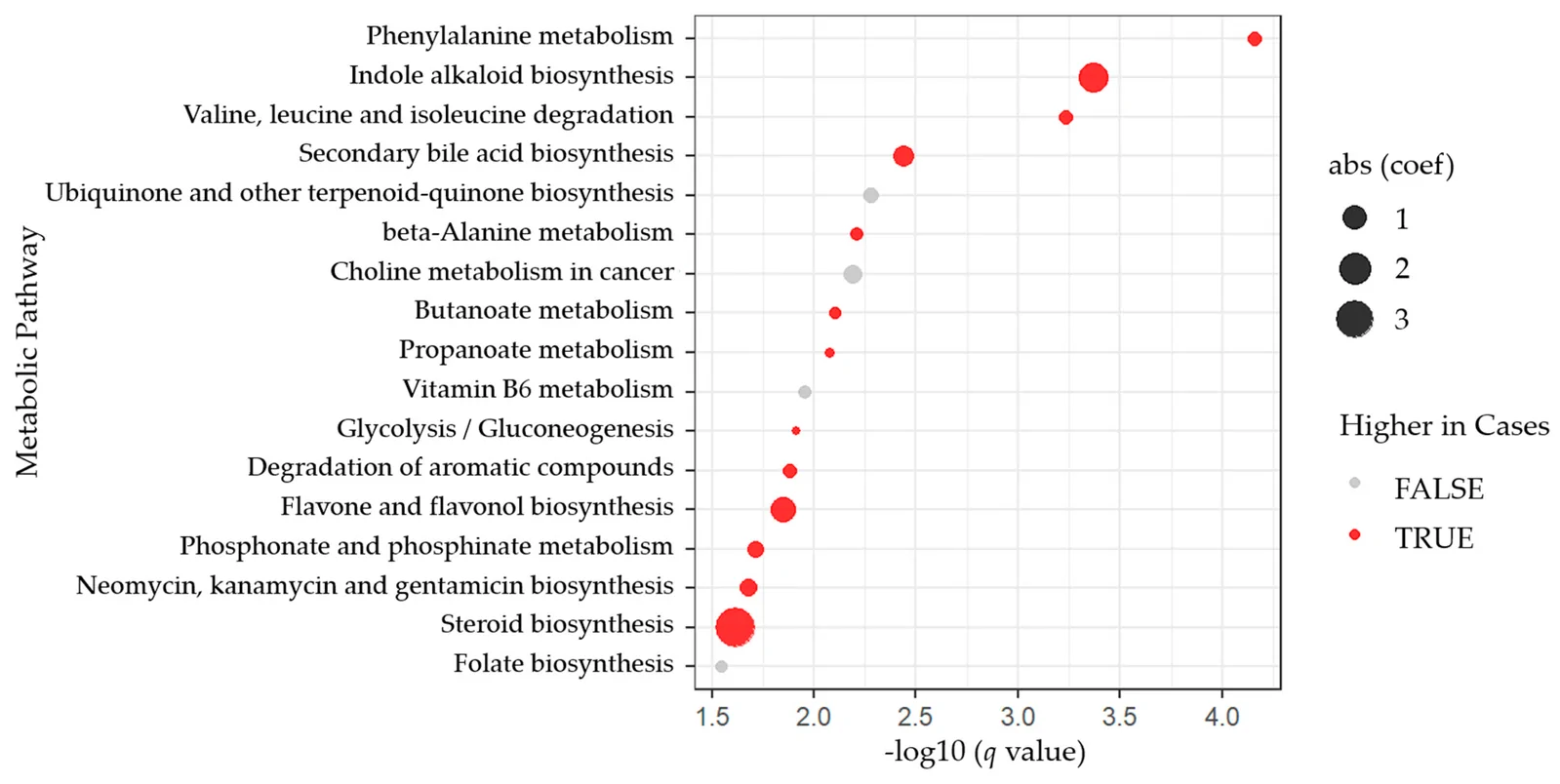
Metabolic pathway enrichment according to cases and controls, adjusted by age, smoking status and body mass index.

Functional profile prediction showed significant enrichment of 111 metabolic pathways associated with SCA status (FDR < 0.05). There were significant differences in predicted pathway enrichment for metabolism of lipids and lipoproteins (e.g., lipoprotein lipase, acylglycerol lipase, lecithin-cholesterol acyltransferase and diacylglycerol kinase), carbohydrates (e.g., glycogenin, 6-phospho-beta-galactosidase, myo-inositol/D-chiro-inositol dehydrogenase) and amino acids (e.g., 5-aminolevulinate synthase, serine O-acetyltransferase and sepiapterin reductase) (Supplementary Table S4). Phenylalanine metabolism and indole alkaloid biosynthesis exhibited the strongest predicted enrichment in a theorical approach derived from microbial abundance and diversity (Figure 6).

## 4. Discussion

In the current study, the upper airway microbiome was described to determine the differences in microbial composition associated with SCA. Our results show lower alpha diversity among SCA cases, accompanied by an enrichment of fermentative and inflammation-modulating microorganisms. These analyses suggest that bacterial communities inhabiting the oropharyngeal mucosa are associated with higher risk of aCVD and may contribute to systemic vascular dysfunction.

Approximately half of the microbiome was represented by *Prevotella*, *Veillonella*, and *Fusobacterium*. *Prevotella* has been proposed to be a sort of constitutive genus in human gut microbiota, with a pivotal role in health and disease status [37]. The relative abundance and diversity of *Prevotella* spp. are known to vary among populations according to inherited and lifestyle-related factors [38]. Accordingly, the relationship of this genus and atherosclerosis-related outcomes is notorious heterogenous. For instance, increased abundance has been associated with higher risk of SCA [39], whereas it has also been linked to lower risk of coronary artery disease [40]. *Veillonella* spp. have been closely related to atherosclerosis at multiples levels of disease development. An early study on microbiomes and atherosclerosis demonstrated that vascular plaques are enriched with *Veillonella* species [41], a finding that has been confirmed by more recent studies, where this genus remains a major component of microbial communities in carotid and coronary atheromas [42,43,44]. Increased abundance of *Veillonella* has also been reported in the oral and gut microbiomes of individuals with atherosclerosis compared with healthy counterparts [14,44,45]. In addition, enrichment of *Veillonella* in subgingival microbiomes has been associated with higher prevalence of type 2 diabetes mellitus [46], while increased abundance of *Veillonella* in the gut microbiome has been associated with lower prevalence of myocardial infarction and higher ventricular ejection fraction [47].

Evidence regarding *Veillonella* enrichment in atherosclerosis-related microbiomes suggests that species of this genus may be transversally involved in aCVD pathogenesis, from early stages by promoting dysbalanced microenvironments to later stages by influencing life-threatening cardiovascular ischemic events. Functionally, the amino acid fermenting capacity of *Veillonella* has been implicated in host adiposity regulation, as amino acid metabolism derives in the production of short-chain fatty acid synthesis and the subsequent secretion of glucagon-like peptide-1 and peptide-YY through activation of Gpr41 and Gpr43 receptors [48,49]. Furthermore, gut *Veillonella* has been thought to contribute to the accumulation of host vascular damage through its involvement in ethanol metabolism, which induces downregulation of the *FANDC* gene, a gene involved cellular repairing pathways [50]. From a pathophysiological perspective, a high abundance of *Veillonella* has been associated with cardiac and vascular functions through promoting Troponina I and N-terminal pro-B-type Natriuretic Peptide release; however, the cellular mechanisms underlying these associations remain unclear [47].

*Fusobacterium* is another highly abundant genus in the human oral and gut microbiome that has been implicated in the pathophysiology of several non-communicable diseases [51]. In particular, *Fusobacterium nucleatum* from the oral cavity has been reported to be more abundant in individuals with SCA, and some authors have proposed biological mechanisms linking this microorganism to disease development through transcriptomic and epigenomic processes associated with autophagy, regulation of inflammatory response and lipid metabolism, among others [51,52]. In our study, *F nucleatum* was more abundant in cases (0.5%) when compared to controls (0.36%); however, this finding requires further explorations to assess potential statistical, biological and clinical significances.

Differential abundances of *Streptococcus*, *Aureimonas*, *Mogibaecterium*, *Candidatus Saccharimonas*, *Pseudomonas*, *Segatella*, *Stomatobaculum*, *Oribacterium* and *Lachnoanaerobaculum* genus were identified when the adjusted MaAsLin2 analysis was applied. For all these taxa, relative abundance was lower in cases than in controls, consistent with the reduced microbial diversity observed among cases based on the Simpson, Shannon and Chao1 diversity indices. Typically, reduction of microbial diversity is regarded as a hallmark of dysbiosis. A recent analysis with oral microbiome data from the National Health and Nutrition Examination Survey (NHANES), revealed that decreased microbial richness was associated with higher all-cause and CVD mortality [53]. Another study on atherosclerosis in patients with obstructive sleep apnea demonstrated an inverse correlation between gut microbiome Shannon index and carotid intima-media thickness [54].

As noted above, our analysis of differences in genus-level relative abundance showed that *Streptococcus* was significantly more abundant in controls than in SCA cases (1.49% vs. 0.64%, respectively). This finding differs from that of most previous studies of oral and gut microbiota, in which several *Streptococcus* species, including *Streptococcus anginosus*, *Streptococcus oralis subsp. oralis*, *Streptococcus parasanguinis*, *Streptococcus gordonii*, and *Streptococcus agalactiae*, have been reported to be enriched in individuals with atherosclerosis [55]. Experimental and clinical evidence has suggested several biological mechanisms linking these species to vascular disease, including translocation into the systemic circulation following oral mucosal disruption, endothelial adhesion, endothelial cell invasion, and persistence within the subendothelial space [55,56,57,58]. At first glance, our findings appear to be at variance with these proposed mechanisms. However, species-level analyses revealed a heterogeneous response within the genus. Species previously associated with vascular disease, including *S. sanguinis*, *S. vestibularis*, and *S. oralis*, also showed lower relative abundance in SCA cases, whereas other streptococcal species exhibited stable or increased abundance. These observations suggest that the overall reduction of the genus reflects species-specific ecological shifts rather than a uniform response across all *Streptococcus* taxa, highlighting the importance of interpreting genus-level associations with caution.

*Aureimonas* was absent in cases, while it represented 1.6% of the bacterial genera identified in controls, which was also reflected in the MaAsLin2 analysis. Members of this genus have been reported as components of the circulating blood microbiome [59] and have been associated with several non-communicable diseases [59,60,61], although no virulence factors directly involved in atherosclerosis pathophysiology have been described to date. In a study on gestational diabetes, *Aureimonas altamirensis* was consistently associated with higher serum glucose, and modulation of the systemic inflammatory response was proposed as a potential underlying mechanism [62]. Further studies investigating the interactions between this genus, vascular cells, and factors of cardiometabolic homeostasis may contribute to clarify whether immune evasion or virulence-related activity are behind plausible host–microbiome interaction.

*Pseudomonas* was another genus that differed significantly in relative abundance between cases and controls. Consistent with the pattern observed for several other genera, *Pseudomonas* was less abundant in SCA cases than in controls (1.9% vs. 11.2%, respectively). This finding is particularly noteworthy because *Pseudomonas* is recognized as one of the predominant genera in the healthy airway microbiome. Although previous studies have more frequently associated the phylum Proteobacteria/Pseudomonadota or specific *Pseudomonas* species with cardiovascular disease, the biological role of this genus appears to dependent strongly on the microbial niche and disease context [63]. Whether the marked reduction in *Pseudomonas* abundance observed in individuals with SCA reflects an early feature of airway dysbiosis or represents a microbial signature associated with SCA remains uncertain and warrants further investigation as longitudinal data from the CaReS cohort become available.

Enriched phenylalanine metabolism in the gut microbiome has been associated with increased serum levels of phenylacetylglutamine, a microbial-derived metabolite known to be a risk factor for cardiovascular complications in subjects with aCVD [64,65]. Previous studies have demonstrated that circulating phenylacetylglutamine promotes macrophage M1 polarization and impairs the transition from an inflammatory to a reparative phenotype through mechanisms involving NF-kB activation and p65 phosporilation [66]. In our analysis, phenylalanine metabolism showed the strongest predicted enrichment. Although this finding is based on a predictive approach, the microbial abundance and diversity observed in the current study suggests a potential mechanism underlying the association with SCA. Further studies incorporating measurements of circulating phenylacetylglutamine might be pertinent to clarify the effects of this pathway on SCA development.

Indole alkaloids constitute a large group of compounds derived from tryptophan and dimethylallyl phosphate that possess well-described anti-atherosclerotic properties. In animal models, serum concentrations of these molecules have been shown to increase in the presence of microbial activity, and their protective effect against atherosclerosis-associated endothelial dysfunction has been reported to be exerted through the ApoE pathway [67,68]. In another murine model, total indole alkaloids extracted from plants (*Alstonia scholaris*) attenuated the fatty liver disease phenotype, including a reduction of hepatic lipid droplet accumulation, through regulation of fatty acid metabolism and oxidative pathways [69]. As with phenylalanine metabolism, conclusions regarding indole alkaloids should be interpreted with caution, since functional predictions from *16SRNA* sequencing and MaAsLin2-based inferences have inherent limitations. Nevertheless, further research on the effects of these compounds on atherosclerosis development may be warranted. Several limitations should be considered when interpreting our results. SCA is a dynamic phenomenon characterized by recurrent progression and regression throughout its natural history. Likewise, dysbiosis is a time-dependent status, with microbial abundance and diversity continuously changing in response to environmental factors. Because of its cross-sectional design, the current study was unable to capture time-related changes in either disease condition or microbiome composition. Therefore, whether the upper airway microbiome influences the SCA trajectories cannot be determined from the present set of data.

Another important limitation relates to sequencing batch effects. Most SCA cases and controls were processed in separate sequencing runs, preventing the independent effects of technical variation from being fully disentangled from the underlying biological differences between study groups. In addition, the significant PERMDISP and Tukey’s HSD results indicate greater within-group dispersion among SCA cases, suggesting that differences in community heterogeneity may have contributed to the observed association between SCA and microbiome composition. To reduce the likelihood that the beta-diversity results were driven primarily by technical artifacts or dispersion effects, we performed a series of complementary sensitivity analyses, including adjusted PERMANOVA, constrained dbRDA, and stratified analyses according to age and LDL cholesterol status. The consistency of these findings across different analytical approaches supports the robustness of the observed association, although the potential influence of batch effects and heterogeneous dispersion cannot be completely excluded. Naturally, future studies should prioritize balanced group characteristics and sequencing designs, reducing case–control discrepancies and potential batch-related confounding effects.

Sample size is another limitation that should be considered. Effect sizes derived from association analyses should be interpreted cautiously, as some microbiome–SCA relationships may not have been detected, whereas others might have been under- or over-estimated. Despite efforts to reduce dissimilarities between case and control groups, age matching was not feasible. Since SCA is more prevalent among older adults, the age range of cases was notoriously narrower, making adequate matching difficult when other cardiometabolic variables and sequencing outputs availability were taken into account. In this regard, we have previously reported an strong association between aging and the prevalence of cardiometabolic disorders in this population [31,70,71,72,73], which made it difficult to identify a metabolically healthy group of subjects over 60 years. To mitigate the impact of this age difference, statistical inferences were adjusted for age and other relevant covariates. Accordingly, the associations between microbiome composition and SCA appeared to be independent of age. Despite these limitations, we believe that our findings provide an interesting perspective on the complexity and dynamics of the oropharyngeal microbiome, highlighting its potential relationship with atherosclerosis and underscoring the need for longitudinal studies to capture extrinsic and shared influences over time. In conclusion, this study provides exploratory evidence on upper airway microbiome variations in diversity associated with SCA. The associations between microbiome and SCA were consistent across multiple sensitivity analyses, including covariate-adjusted PERMANOVA and constrained distance-based redundancy analysis; however, they should be interpreted with caution, since unequal multivariate dispersion, and the potential confounding effect attributable to sequencing batch remains and might be influencing the observed differences. Further studies with a larger study cohort, balanced sequencing designs, and longitudinal approaches would be pertinent to better elucidate the potential role of the airway microbiome in the development of SCA.

## Figures and Tables

**Table 1:** Demographic, socioeconomic and clinical characteristics for cases and controls.

Variable	Cases (*n* = 40)	Controls (*n* = 40)	*p*-Value
Age, y.o., mean (SD)	60.08 (8.44)	55.50 (8.43)	0.018
Sex, % (*n*)			
Women	42.5 (17)	30.0 (12)	
Men	57.5 (23)	70 (28)	0.352
Income, USD, median [IQR]	268.3 [146.3; 512.2]	243.9 [146.3; 664.6]	0.95
Smoking status, % (*n*)			
Never	71.4 (36)	81.2 (41)	
Former	22.0 (11)	14.5 (7)	
Current	6.5 (3)	4.3 (1)	0.44
Body mass index, Kg/m^2^, mean (SD)	29.3 (4.94)	28.1 (4.55)	0.242
Pulmonary function (post-bronchodilator), L, mean (SD)			
FVC	2.93 (0.78)	2.78 (0.62)	0.336
FEV_1_	2.46 (0.62)	2.42 (0.56)	0.797
FEV_1_/FVC	84.23 (3.72)	87.29 (3.73)	<0.001
Cardiometabolic parameters, mean (SD)			
Systolic blood pressure, mmHg	130.28 (18.25)	127.26 (19.65)	0.481
Diastolic blood pressure, mmHg	78.60 (10.78)	80.18 (10.58)	0.513
Fasting glycemia, mg/dL	104.81 (27.33)	103.00 (53.76)	0.850
Serum total cholesterol, mg/dL	195.85 (43.38)	217.95 (54.10)	0.047
Serum LDLc, mg/dL	122.46 (40.52)	144.62 (48.04)	0.029
Triglyceridemia, mg/dL	131.32 (64.86)	135.80 (98.33)	0.810
Serum HDLc, mg/dL	47.12 (12.47)	46.17 (12.16)	0.731
Carotid intima-media thickness, mm, mean (SD)	1.03 (0.15)	0.65 (0.11)	<0.001

## Data Availability

The data presented in the study are openly available in FASTQ data Upper Airway—Cartagena Cohort Study at https://doi.org/10.5281/zenodo.20683915, reference number md5:bc82d22b69b7b450d0b285147c6ff3bf.

## Supplementary Materials

The following supporting information can be downloaded at https://www.mdpi.com/article/10.3390/microorganisms14081699/s1, Figure S1: Rarefaction curves for cases and controls; Table S1: Sequencing depth per sample; Table S2: Abundance and relative abundance at genus level for cases and controls; Table S3: Abundance and relative abundance at species level for cases and controls; Table S4: Enriched metabolic pathways associated with subclinical atherosclerosis.
